# Telemedicine to deliver diabetes care in low- and middle-income countries: a systematic review and meta-analysis

**DOI:** 10.2471/BLT.19.250068

**Published:** 2020-11-29

**Authors:** Jorge César Correia, Hafsa Meraj, Soo Huat Teoh, Ahmed Waqas, Maaz Ahmad, Luis Velez Lapão, Zoltan Pataky, Alain Golay

**Affiliations:** a Department of Medicine, Geneva University Hospitals, Chemin Venel 7, 1206 Geneva, Switzerland.; b Faculty of Life Sciences and Education, University of South Wales, Pontypridd, Wales.; c Advanced Medical and Dental Institute, Universiti Sains Malaysia, Penang, Malaysia.; d Institute of Population Health, University of Liverpool, Liverpool, England.; e Department of Oral Biology, Sharif Medical and Dental College, Lahore, Pakistan.; f Instituto de Higiene e Medicina Tropical, Universidade Nova de Lisboa, Lisbon, Portugal.

## Abstract

**Objective:**

To determine the effectiveness of telemedicine in the delivery of diabetes care in low- and middle-income countries.

**Methods:**

We searched seven databases up to July 2020 for randomized controlled trials investigating the effectiveness of telemedicine in the delivery of diabetes care in low- and middle-income countries. We extracted data on the study characteristics, primary end-points and effect sizes of outcomes. Using random effects analyses, we ran a series of meta-analyses for both biochemical outcomes and related patient properties.

**Findings:**

We included 31 interventions in our meta-analysis. We observed significant standardized mean differences of −0.38 for glycated haemoglobin (95% confidence interval, CI: −0.52 to −0.23; *I*^2^ = 86.70%), −0.20 for fasting blood sugar (95% CI: −0.32 to −0.08; *I*^2^ = 64.28%), 0.81 for adherence to treatment (95% CI: 0.19 to 1.42; *I*^2^ = 93.75%), 0.55 for diabetes knowledge (95% CI: −0.10 to 1.20; *I*^2^ = 92.65%) and 1.68 for self-efficacy (95% CI: 1.06 to 2.30; *I*^2^ = 97.15%). We observed no significant treatment effects for other outcomes, with standardized mean differences of −0.04 for body mass index (95% CI: −0.13 to 0.05; *I*^2^ = 35.94%), −0.06 for total cholesterol (95% CI: −0.16 to 0.04; *I*^2^ = 59.93%) and −0.02 for triglycerides (95% CI: −0.12 to 0.09; *I*^2^ = 0%). Interventions via telephone and short message service yielded the highest treatment effects compared with services based on telemetry and smartphone applications.

**Conclusion:**

Although we determined that telemedicine is effective in improving several diabetes-related outcomes, the certainty of evidence was very low due to substantial heterogeneity and risk of bias.

## Résumé

**Objectif:**

Mesurer l'efficacité de la télémédecine dans la prise en charge du diabète au sein des pays à faible et moyen revenu.

**Méthodes:**

Nous avons examiné sept bases de données, la plus récente datant de juillet 2020, en quête d'essais cliniques randomisés s'intéressant à l'efficacité de la télémédecine dans les soins prodigués aux patients diabétiques résidant dans des pays à faible et moyen revenu. Nous y avons trouvé des informations sur les caractéristiques de l'étude, les principaux critères d'évaluation et l'ampleur des effets pour les résultats obtenus. Grâce à des modèles à effets aléatoires, nous avons mené une série de méta-analyses sur les résultats biochimiques et les propriétés des patients concernés.

**Résultats:**

Notre méta-analyse a tenu compte de 31 interventions. Nous avons observé d'importantes différences moyennes standardisées: −0,38 pour l'hémoglobine glyquée (intervalle de confiance de 95%, IC: −0,52 à −0,23; *I*^2^ = 86,70%), −0,20 pour la glycémie à jeun (IC de 95%: −0,32 à −0,08; *I*^2^ = 64,28%), 0,81 pour l'adhésion au traitement (IC de 95%: 0,19 à 1,42; *I*^2^ = 93,75%), 0,55 pour la connaissance de la maladie (IC de 95%: 0,10 à 1,20; *I*^2^ = 92,65%) et 1,68 pour l'auto-efficacité (IC de 95%: 1,06 à 2,30; *I*^2^ = 97,15%). Nous n'avons constaté aucune répercussion significative du traitement sur d'autres résultats, avec des différences moyennes standardisées de −0,04 pour l'indice de masse corporelle (IC de 95%: −0,13 à 0,05; *I*^2^ = 35,94%), de −0,06 pour le cholestérol total (IC de 95%: −0,16 à 0,04; *I*^2^ = 59,93%) et de −0,02 pour les triglycérides (IC de 95%: −0,12 à 0,09; *I*^2^ = 0%). Ce sont les interventions par téléphone et par SMS qui ont eu le plus d'impact sur le traitement par rapport aux services recourant à la télémétrie et aux applications sur smartphone.

**Conclusion:**

Bien que nous ayons établi l'efficacité de la télémédecine dans l'amélioration de nombreux résultats liés au diabète, le degré de certitude s'est révélé très bas en raison d'une grande hétérogénéité et du risque de biais.

## Resumen

**Objetivo:**

Determinar la eficacia de la telemedicina en la prestación de servicios de atención sanitaria de la diabetes en los países con ingresos bajos y pocos medios.

**Métodos:**

Se realizaron búsquedas en siete bases de datos hasta julio de 2020 para encontrar ensayos controlados aleatorios que investigaran la eficacia de la telemedicina en la prestación de servicios de atención de la diabetes en países con ingresos bajos y pocos medios. Se extrajeron datos sobre las características del estudio, las principales variables de evaluación y los tamaños del efecto de los resultados. Utilizando el análisis de los efectos aleatorios, realizamos una serie de metaanálisis tanto para los resultados bioquímicos como para las propiedades relacionadas con los pacientes.

**Resultados:**

Incluimos 31 intervenciones en nuestro metaanálisis. Se observaron diferencias medias estandarizadas significativas de -0,38 para la hemoglobina glicosilada (intervalo de confianza del 95%, IC: -0,52 a -0,23; I^2^ = 86,70%), -0,20 para la glucemia en ayunas (IC del 95%: -0,32 a -0,08; I^2^ = 64,28%), 0,81 para el cumplimiento del tratamiento (IC del 95%: 0,19 a 1,42; I^2^ = 93,75%), 0,55 para el conocimiento de la diabetes (IC del 95%: 0,10 a 1,20; I^2^ = 92,65%) y 1,68 para la autoeficacia (IC del 95%: 1,06 a 2,30; I^2^ = 97,15%). No se observaron efectos significativos del tratamiento para otros resultados, con diferencias medias estandarizadas de -0,04 para el índice de masa corporal (IC del 95%: -0,13 a 0,05; I^2^ = 35,94%), -0,06 para el colesterol total (IC del 95%: -0,16 a 0,04; I^2^ = 59,93%) y -0,02 para los triglicéridos (IC del 95%: -0,12 a 0,09; I^2^ = 0%). Las intervenciones telefónicas y el servicio de mensajes cortos de texto produjeron los efectos de tratamiento más altos en comparación con los servicios basados en la telemetría y las aplicaciones de los teléfonos inteligentes.

**Conclusión:**

Aunque se determinó que la telemedicina es eficaz para mejorar varios resultados relacionados con la diabetes, la certeza de las pruebas fue muy baja debido a la considerable heterogeneidad y el riesgo de sesgo.

## ملخص

الغرض تحديد مدى فعالية العلاج الطبي عن بعد في تقديم الرعاية لمرض السكري في البلدان منخفضة الدخل والبلدان متوسطة الدخل.

الطريقة قمنا بالبحث في سبع قواعد للبيانات حتى يوليو/تموز 2020، عن تجارب معشاة مضبطة بالشواهد للتحقق من فعالية العلاج الطبي عن بعد في تقديم الرعاية لمرض السكري في البلدان منخفضة الدخل والبلدان متوسطة الدخل. واستخلصنا البيانات حول خصائص الدراسة، ونقاط النهاية الأولية، وأحجام تأثير النتائج. وباستخدام تحليلات التأثيرات العشوائية، قمنا بإجراء سلسلة من التحليلات التلوية لكل من النتائج الحيوية الكيميائية، والخصائص المتعلقة بالمريض.

النتائج قمنا بتضمين 31 تدخلاً في تحليلنا التلوي. لاحظنا متوسطات فروق معيارية ملموسة بقيمة -0.38 في الهيموجلوبين السكري (فاصل الثقة 95%: -0.52 إلى -0.23؛ / ^2 ^)، -0.20 لسكر الدم أثناء الصيام (فاصل الثقة 95‏%: -0.32 إلى -0.08؛ / ^2 ^)، 0.81 للتمسك بالعلاج (فاصل الثقة 95‏%: 0.19 إلى 1.42؛ / ^2 ^)، 0.55 للإلمام بمرض السكري (فاصل الثقة 95‏%: 0.10 إلى 1.20؛ / ^2 ^)، و1.68 للفعالية الذاتية (فاصل الثقة 95‏%: 1.06 إلى 2.30؛ / ^2 ^ = 97.15%). لم نلحظ أي آثار ملموسة للعلاج بالنسبة للنتائج الأخرى، مع متوسطات فروق معيارية قدرها -0.04 لمؤشر كتلة الجسم (فاصل ثقة 95%: -0.13 إلى 0.05؛ / ^2 ^ = 35.94%)، -0.06 للكوليسترول الكلي (فاصل الثقة 95‏%: -0.16 إلى 0.04؛ / ^2 ^ = 59.93%)، -0.02 للدهون الثلاثية (فاصل الثقة 95‏%: -0.12 إلى 0.09؛ / ^2 ^ = 0%). حققت التدخلات عبر الهاتف وخدمة الرسائل القصيرة أعلى تأثيرات للعلاج مقارنة بالخدمات القائمة على تطبيقات القياس عن بعد والهواتف الذكية.

الاستنتاج على الرغم من أننا قد قررنا أن العلاج الطبي عن بعد فعال في تحسين العديد من النتائج المتعلقة بمرض السكري، إلا أن الثقة في الأدلة كانت منخفضة للغاية بسبب عدم التجانس الموضوعي وخطر التحيز. ****

## 摘要

**目的:**

探讨远程医疗在中低收入国家糖尿病护理方面的应用效果。

**方法:**

截至 2020 年 7 月，我们检索了 7 个数据库，开展随机对照试验以研究远程医疗在中低收入国家糖尿病护理方面的应用效果。我们提取了与研究特征、主要终点和结果效应值有关的数据。通过使用随机效应分析，我们针对生化结果和相关患者属性进行了一系列荟萃分析。

**结果:**

我们将 31 项干预措施纳入了荟萃分析。我们观察到显著的标准化均数差，具体地，糖化血红蛋白的标准化均数差为0.38（95% 置信区间，CI：0.52 至 0.23；*I*^2 ^= 86.70%），空腹血糖的标准化均数差为0.20 （95% 置信区间：0.32 至 0.08；*I*^2^= 64.28%），坚持治疗率的标准化均数差为 0.81（95% 置信区间：0.19 至 1.42；*I*^2^ = 93.75%），糖尿病知识普及的标准化均数差为 0.55（95% 置信区间：0.10 至 1.20；*I*^2^ = 92.65%）且自我效能感的标准化均数差为 1.68（95% 置信区间：1.06 至 2.30；*I*^2^ = 97.15%）。根据我们的观察，其他结果没有表现出明显的治疗效果，具体地，身体质量指数的标准化均数差为0.04（95% 置信区间：0.13 至 0.05；*I*^2 ^= 35.94%），总胆固醇的标准化均数差为0.06（95% 置信区间：0.16 至 0.04；*I*^2^ = 59.93%）且甘油三酯的标准化均数差为0.02（95% 置信区间：0.12 至 0.09；*I*^2^= 0%）。与基于遥测技术和智能手机应用的服务相比，通过电话和短信服务进行干预所取得的治疗效果最佳。

**结论:**

虽然我们确定远程医疗可以有效地改善一些糖尿病引起的症状，但由于存在严重的异质性和偏差风险，证据确定性非常低。

## Резюме

**Цель:**

Определить эффективность телемедицины для оказания медицинской помощи при диабете в странах с низким и средним уровнем доходов.

**Методы:**

Авторы провели поиск по семи базам данных до июля 2020 года на предмет рандомизированных контролируемых исследований, посвященных эффективности телемедицины как способа оказания помощи при диабете в странах с низким и средним уровнем доходов. Были извлечены данные о характеристиках исследования, основных конечных точках и величинах эффекта в результатах. Используя анализ случайных эффектов, авторы выполнили серию метаанализов как биохимических результатов, так и связанных с ними свойств пациентов.

**Результаты:**

В метаанализ был включен 31 случай вмешательства. Авторы наблюдали значительную стандартизованную разницу средних значений, составившую -0,38 для гликированного гемоглобина (95%-й ДИ: -0,52–-0,23; *I*^2^ = 86,70%), -0,20 для уровня сахара крови натощак (95%-й ДИ: -0,32–-0,08; *I*^2^ = 64,28%), 0,81 для соблюдения режима лечения (95%-й ДИ: 0,19–1,42; *I*^2^ = 93,75%), 0,55 для осведомленности о диабете (95%-й ДИ: 0,10–1,20; *I*^2^ = 92,65%) и 1,68 для уверенности в собственных силах (95%-й ДИ: 1,06–2,30; *I*^2^ = 97,15%). Не наблюдалось значительных эффектов лечения для других результатов со стандартизованной разностью средних значений: -0,04 для индекса массы тела (95%-й ДИ: -0,13–0,05; *I*^2^ = 35,94%), -0,06 для общего холестерина (95%-й ДИ: -0,16–0,04; *I*^2^ = 59,93%) и -0,02 для триглицерида (95%-й ДИ: -0,12–0,09; *I*^2^ = 0%). Вмешательства по телефону и посредством службы коротких сообщений дали наивысший лечебный эффект по сравнению с услугами, основанными на телеметрии и приложениях для смартфонов.

**Вывод:**

Хотя было выявлено, что телемедицина эффективна для улучшения некоторых результатов, связанных с диабетом, достоверность доказательств была очень низкой из-за значительной неоднородности и риска систематической ошибки.

## Introduction

Diabetes mellitus is one of the most prevalent chronic and preventable conditions affecting over 415 million people globally, and accounted for over 5 million deaths in 2015.^1^ Because the symptoms of diabetes affect both the micro- and macrovascular systems, the disease is associated with significant morbidity, mortality and a poor quality of life.^2^^,^^3^ By 2030, the estimated annual medical and related costs of both type 1 and type 2 diabetes in the United States of America alone will reach a staggering 622 billion United States dollars (US$).^4^ Over 75% of patients with diabetes live in low- and middle-income countries,^1^ where most patients obtain diabetes treatment only after making out-of-pocket payments. A study reported the costs of diabetes treatment as US$ 7 per visit for an outpatient, US$ 290 per year for an inpatient, and US$ 25 and US$ 177 per patient per year for laboratory and medication costs, respectively.^5^ In sub-Saharan Africa, recent studies estimate the financial burden related to diabetes as US$ 19.5 billion, about 1.2% of the cumulative regional gross domestic product.^6^

The prevalence of diabetes in low- and middle-income countries has risen faster than in high-income countries, with the highest rise observed in the Eastern Mediterranean Region. The prevalence of diabetes is highest in low- and middle-income countries (12.3%) followed by upper-middle-income countries (11.1%), and lowest in high-income countries (6.6%).^7^ This high prevalence in low- and middle-income countries, coupled with a lack of both quality health-care services and equity in health care, means that the long-term management of diabetes is a major global challenge.^1^^,^^8^ It is often opined that these inequalities in diabetes care, as well as inequities in health-care services and slow progress towards achievement of universal health coverage, could be addressed by employing telemedicine-based interventions. 

Telemedicine is the practice by which telecommunication and information technology are used to provide clinical health care to distant patients.^9^ In a broader context, digital health interventions can help stakeholders to overcome several health systems challenges. These challenges include an insufficient supply of commodities, poor adherence to guidelines by health-care professionals, poor adherence to treatment by patients, a lack of access to information or data, and a loss of patients to follow-up. Combined with decision support systems, telemedicine can help by providing protocol checklists, providing prompts and alerts as per protocols, enhancing communication between health-care providers and patients, and compiling the schedules of health-care providers. Telemedicine can also aid in routine health-care data collection by increasing the use of electronic medical records and health management information systems.^10^

Several telemedicine interventions targeting diabetes have been implemented in low- and middle-income countries;^11^^–^^18^ however, evidence synthesis efforts in such settings are scarce. Although studies to evaluate the clinical efficacy or cost–effectiveness of telemedicine interventions for diabetes have previously been conducted,^9^^,^^11^^–^^19^ these reviews were performed over a global context and were dominated by evidence from high-income countries (mostly the USA). Such previous syntheses have little relevance in determining the effectiveness of telemedicine for diabetes treatment in low- and middle-income countries.

In this systematic review and meta-analysis, we aim to address the lack of evidence synthesis efforts in telemedicine for diabetes care in low- and middle-income countries. We designed our study to: (i) estimate the effectiveness of telemedicine in improving biochemical outcomes and patient characteristics such as adherence to treatment and self-efficacy; (ii) evaluate the implementation processes involved in telemedicine interventions; and (iii) determine the certainty of evidence for telemedicine-based interventions for diabetes care in low- and middle-income countries.

## Methods

### Database search

We conducted this systematic review and meta-analysis as per Preferred Reporting Items for Systematic Reviews and Meta-Analyses guidelines (data repository).^20^^,^^21^ We registered the review protocol before at the PROSPERO database for systematic reviews (CRD42019141271).^22^ Using a pre-tested search strategy (data repository),^21^ we searched Web of Science, PubMed®, MEDLINE®, Global Health Library and Cochrane Central Register of Controlled Trials from their inception to August 2019. We also searched New York Academy of Medicine and POPLINE databases for grey literature. We applied no restrictions in terms of participant age, year of publication or region of study at this stage.

We conducted an updated database search up to July 2020. We augmented the previously used search strategy with the keywords health informatics, wireless devices, text messaging, clinical decision support system, mobile app, blood glucose, diabetes mellitus type 2 and T2DM (Box 1; available at: http://www.who.int/bulletin/volumes/99/3/19-250068).

Box 1 **PubMed search strings used in systematic review and meta-analysis of the effectiveness of telemedicine in the delivery of diabetes care, low- and middle-income countries, 2010–2020**

• Telemedicine: ((Telemedicine[Title/Abstract]) OR (“health informatics”[Title/Abstract]) OR (“wireless devices”[Title/Abstract]) OR (“text messag*”[Title/Abstract]) OR (“clinical decision support system”[Title/Abstract]) OR (“mobile app”[Title/Abstract]) OR (tele-health[Title/Abstract]) OR (telerehabilitation[Title/Abstract]) OR (telecommunication[Title/Abstract]) OR (“remote consultation”[Title/Abstract]) OR (“mobile health”[Title/Abstract]) OR (mHealth[Title/Abstract]) OR (eHealth[Title/Abstract]))

• Diabetes: ((Diabetes[Title]) OR (“blood glucose”[Title]) OR (“Diabetes mellitus type 2”[Title]) OR (T2DM[Title]) OR (“Diabetes mellitus”[Title]))

• Trial: ((RCT[Title/Abstract]) OR (trial*[Title/Abstract]) OR (“controlled trial”[Title/Abstract]) OR (“randomized controlled”[Title/Abstract]) OR (“cluster randomized controlled trial”[Title/Abstract]))

• Country: (“Middle income country” OR “Middle income countries” OR “low income countries” OR “low income country” OR LMIC OR “developing world” OR “developing country” OR “developing countries” OR Afghanistan OR “Kyrgyz Republic” OR Bangladesh OR Liberia OR Benin OR Madagascar OR “Burkina Faso” OR Malawi OR Burundi OR Mali OR Cambodia OR Mauritania OR “Central African Republic” OR Mozambique OR Chad OR Myanmar OR Comoros OR Nepal OR Congo OR Niger OR Eritrea OR Rwanda OR Ethiopia OR “Sierra Leone” OR Gambia OR Somalia OR Guinea OR Tajikistan OR Guinea-Bissau OR United Republic of Tanzania OR Haiti OR Togo OR Kenya OR Uganda OR Korea OR Zimbabwe OR Algeria OR Libyan Arab Jamahirya OR American-Samoa OR Lithuania OR Angola OR The former Yugoslav Republic of Macedonia OR Antigua OR Malaysia OR Argentina OR Maldives OR Azerbaijan OR Mauritius OR Belarus OR Mexico OR Bosnia OR Montenegro OR Botswana OR Namibia OR Brazil OR Palau OR Bulgaria OR Panama OR Chile OR Peru OR China OR Romania OR Colombia OR “Russian Federation” OR “Costa Rica” OR Serbia OR Cuba OR Seychelles OR Dominica OR “South Africa” OR “Dominican Republic” OR “St Lucia” OR Ecuador OR “St Vincent” OR “The Grenadines” OR Gabon OR Suriname OR Grenada OR Thailand OR “Iran Islamic Republic” OR Islamic Republic of Iran OR Tunisia OR Jamaica OR Turkey OR Jordan OR Turkmenistan OR Kazakhstan OR Tuvalu OR Latvia OR Uruguay OR Lebanon OR Venezuela OR Albania OR the Republic of Moldova OR Armenia OR Mongolia OR Belize OR Morocco OR Bhutan OR Nicaragua OR Bolivia OR Nigeria OR Cameroon OR Pakistan OR “Cape Verde” OR “Papua New Guinea” OR “Congo Republic” OR Paraguay OR “Cote d Ivoire” OR Philippines OR Djibouti OR Samoa OR “Egypt Arab Republic” OR Egypt OR “Sao Tome” OR “El Salvador” OR Senegal OR Fiji OR “Solomon Islands” OR Georgia OR “South Sudan” OR Ghana OR “Sri Lanka” OR Guatemala OR Sudan OR Guyana OR Swaziland OR Honduras OR “Syrian Arab Republic” OR India OR “Timor Leste” OR Indonesia OR Tonga OR Iraq OR Ukraine OR Kiribati OR Uzbekistan OR Kosovo OR Vanuatu OR “Lao PDR” OR Viet Nam OR Lesotho OR “West Bank” OR Gaza OR “Marshall Islands” OR “Yemen Republic” OR Federated States of Micronesia OR Zambia)

Note: Country names are reported here as in the authors’ search string, and have not been edited as per the naming standards of the World Health Organization. Quotation marks are required for multiword search items.

### Inclusion and exclusion criteria

We included telemedicine interventions encompassing various modes of delivery including, but not limited to, short message service (SMS), smartphone applications (apps), telemetry (devices that allow remote monitoring of health data by automatic transmission from patients to clinicians), telephone and web-based systems. We only included randomized controlled trials (RCTs) and cluster RCTs that tested the effectiveness of telemedicine-based interventions in type 1, type 2 and gestational diabetes. We included studies conducted among participants aged ≥ 18 years resident in low- and middle-income countries. We considered all studies that reported serum glycated haemoglobin (HbA1c) levels as either their primary or secondary outcomes; however, to formulate a clinical recommendation for telemedicine interventions, we considered HbA1c levels as a primary outcome in our review.

We excluded studies employing semi-experimental designs, such as pre–post studies or those lacking control groups. In the case where two separate studies were based on an overlapping data set, we only included the study with the most complete information. We excluded short papers, brief reports, abstracts, conference papers, posters and letters to editors because these types of publications often lack important quantitative information. We also excluded studies published in languages other than English because of our lack of translational resources.

### Outcome choice

We included biochemical parameters such as body mass index (BMI) and serum levels of fasting blood sugar, HbA1c, total cholesterol and triglycerides. We also included non-biochemical characteristics such as adherence to treatment, knowledge of diabetes and self-efficacy. However, our primary outcome was HbA1c levels reported post-intervention. 

### Screening process

Two independent reviewers performed the screening process to retrieve bibliographic records of eligible studies in two phases. First, the reviewers screened the titles and abstracts of all bibliographic records against the inclusion and exclusion criteria. Second, the reviewers thoroughly read the full texts of these eligible titles to ensure that all inclusion and exclusion criteria were met. Studies judged to be eligible at this stage were then included in the qualitative and quantitative synthesis where applicable. In the case of any uncertainty or difference in opinion between the reviewers, the reviewers and first author reached a final decision through discussion.

### Data extraction

We extracted data related to the characteristics of the studies, primary end-points and effect sizes of outcomes. We also compiled data on intervention implementation processes, such as mode of delivery, theoretical orientation, rationale, materials, and development and training procedures. We closely examined the individual elements of the interventions according to the World Health Organization guidelines on digital interventions.^10^ We assessed the risk of bias in the RCTs using the Cochrane tool against randomization, allocation concealment, blinding of outcome assessors, attrition rate, selective reporting and any other bias matrices (e.g. a priori protocol registration and statistical power).^23^ It was not possible to assess the rigour of the blinding procedures of participants and personnel in this review because of the nature of telemedicine-based interventions. We assessed certainty of evidence for telemedicine-based interventions using the grading of recommendations, assessment, development and evaluations (GRADE) guidelines.^24^ We conducted the grading of evidence only for the HbA1c end-point as this was our primary outcome. We downgraded evidence by 1 or 2 points according to the presence and extent of flaws such as bias, inconsistency, publication bias, imprecision or indirectness related to patients, outcomes and interventions.^24^

For quantitative synthesis, we assessed the mean and the standard deviation (SD) of outcomes for both the intervention and active control groups.^25^ We used categorical data, such as frequency of events and sample size, for outcomes that lacked quantitative data in the form of mean and SD.^25^ We then used these raw data to calculate standardized mean differences and their SDs for each outcome reported in the included studies. Because of the expected methodological and statistical heterogeneity, we calculated pooled effect sizes by performing random effects analyses.^25^ We present these pooled effect sizes as forest plots depicting standardized mean differences and 95% confidence intervals (CIs). We ran a sensitivity analysis using the single-study knockout approach to assess the contribution of each study to the pooled effect size. We assessed publication bias by creating Begg funnel plots and performing Egger regression analyses (considered significant at *P* ≤ 0.1).^25^ For outcomes demonstrating significant publication bias, we calculated adjusted standardized mean differences using the Duval and Tweedie trim-and-fill method.^25^ For each outcome, we performed a series of subgroup analyses to quantify the specific difference in effect sizes for each mode of delivery.^26^

## Results

### Intervention characteristics

**Fig. 1:**
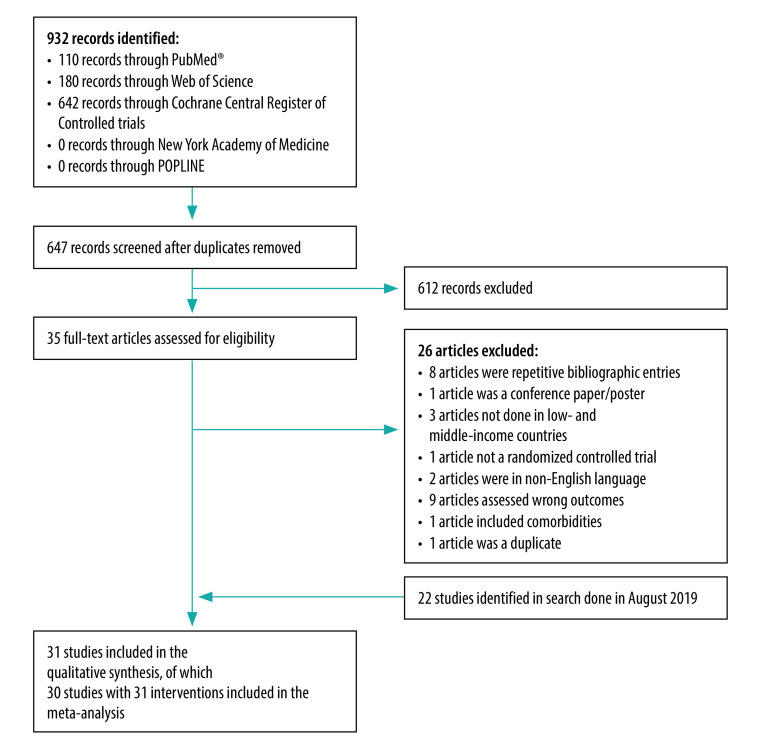
Flowchart of study selection process in the systematic review and meta-analysis of the effectiveness of telemedicine in the delivery of diabetes care, low- and middle-income countries, July 2020

Notes: The database Web of Science includes MEDLINE®. We did not include the Global Health Library, because it had been discontinued at the time of the search. The flowchart for the search done in August 2019 is available in the data repository.^21^

Out of 376 studies retrieved in our initial database search, we included a total of 22 studies describing 23 interventions in the qualitative analysis, and 21 studies describing 22 interventions in the quantitative synthesis (data repository).^21^ In our updated database search, we retrieved 647 non-duplicate bibliographic records; we included 31 of these studies describing 32 interventions (an increase of nine compared with our initial search) in our synthesis (Fig. 1). All included studies were published between 2010 and 2020. A breakdown of the studies by country revealed that the highest number of studies were conducted in China (eight studies), followed by India (five studies), the Islamic Republic of Iran (five studies), Malaysia (three studies), Turkey (two studies) and South Africa (two studies). A single RCT was conducted in each of Brazil, Egypt, Mexico, Mongolia and Pakistan. One of the studies described an intervention conducted at several sites in Cambodia, Congo and the Philippines. The minimum sample size of the RCTs ranged from 60^15^^,^^27^^,^^28^ to 3324.^14^ We list the properties of the 31 studies in Table 1, and the values for the outcomes considered that were extracted from the studies in the data repository.^21^

**Table 1:** Characteristics of included studies in the systematic review and meta-analysis of the effectiveness of telemedicine in the delivery of diabetes care, low- and middle-income countries, 2010–2020

Author, year	Country	Primary end-point for outcome	Mean age (years)	Mode of delivery of intervention	Outcomes analysed
Nesari et al., 2010^29^	Iran (Islamic Republic of)	12 weeks	51.9	Telephone follow-ups	Adherence to treatment, HbA1c
Shetty et al., 2011^30^	India	1 year	50.1	SMS reminders	BMI, HbA1c, total cholesterol, triglycerides
Zhou et al., 2014^31^	China	3 months	NR	Web-based diabetes telemedicine system	BMI, fasting blood sugar, HbA1c, total cholesterol, triglycerides
Shahid et al., 2015^18^	Pakistan	4 months	48.95	Telephone follow-ups	BMI, HbA1c
Anzaldo-Campos et al., 2016^32^	Mexico	4 months	51.5	SMS, educational videos and online brochures	BMI, diabetes knowledge, HbA1c, total cholesterol, triglycerides
Aytekin Kanadli et al., 2016^33^	Turkey	3 months	NR	Telephone follow-ups	BMI, HbA1c, self-efficacy, total cholesterol, triglycerides
Gopalan et al., 2016^34^	South Africa	NR	NR	Educational emails	Qualitatively analysed dietary-consumption-related outcomes
Kim et al., 2016^35^	China	3 months	NR	Internet-based glucose monitoring system	BMI, fasting blood sugar, HbA1c, total cholesterol, triglycerides
Peimani et al., 2016^13^	Iran (Islamic Republic of)	3 months	49.78	Educational SMS and telephone follow-ups	BMI, fasting blood sugar, HbA1c, self-efficacy, total cholesterol, triglycerides
Abaza & Marschollek, 2017^36^	Egypt	3 months	51.24	SMS and reminder prompts	Adherence to treatment, diabetes knowledge, HbA1c, self-efficacy
Kleinman et al., 2017^37^	India	3 months	48.4	Smartphone app and web portal	Adherence to treatment, BMI, fasting blood sugar, HbA1c
Lee et al., 2017^38^	Malaysia	NR	53.24	Smartphone app	BMI, diabetes knowledge, fasting blood sugar, HbA1c, self-efficacy
Hemmati Maslakpak et al., 2017^27^	Iran (Islamic Republic of)	3 months	49.46	Educational telephone calls	Adherence to treatment, BMI, HbA1c, fasting blood sugar, self-efficacy, total cholesterol, triglycerides
Namjoo Nasab et al., 2017^15^	Iran (Islamic Republic of)	NR	NR	Telephone follow-ups	Adherence to treatment, BMI
Van Olmen et al., 2017^39^	Cambodia, Congo, Philippines	1 year	NR	SMS and voice messages	Diabetes knowledge, HbA1c
Wang et al., 2017^40^	China	3 months	52.6	U-Healthcare website, website messages, telephone prompt	BMI, fasting blood sugar, HbA1c, total cholesterol, triglycerides
de Vasconcelos et al., 2018^41^	Brazil	24 weeks	60.9	Guidance and coaching via telephone calls from research nurse	BMI, HbA1c, high-density lipoproteins, total cholesterol, triglycerides
Fang & Deng, 2018^42^	China	3 months	57.69	SMS	BMI, fasting blood sugar, HbA1c, total cholesterol, triglycerides
Ramadas et al., 2018^43^	Malaysia	6 months	49.6	Web-delivered intervention program, log-in reminders via email and text message follow-ups	Diabetes knowledge, fasting blood sugar, HbA1c
Sarayani et al., 2018^44^	Iran (Islamic Republic of)	3 months	53.4	Telephone-based consultation	Adherence to treatment, HbA1c, total cholesterol, triglycerides
Duruturk & Özköslü, 2019^45^	Turkey	6 weeks	52.82	Video conference consultation	HbA1c
Goruntla et al., 2019^46^	India	6 months	58.8	SMS and reminder prompts	Adherence to treatment, BMI, HbA1c, triglycerides
Guo et al., 2019^47^	China	13 weeks	31.2	D-nurse app for monitoring of blood glucose levels and transmission of data to clinic	HbA1c
Prabhakaran et al., 2019^14^	India	1 year	56.7	Android app and SMS reminders	BMI, fasting blood sugar HbA1c, total cholesterol
Sun et al., 2019^48^	China	3 months	67.9	Web-based app, SMS reminders, telephonic reminders	BMI, fasting blood sugar, HbA1c, total cholesterol, triglycerides
Vinitha et al., 2019^49^	India	24 months	43.3	SMS to reinforce healthy lifestyle practices and adherence to medication	BMI, HbA1c, high-density lipoproteins, fasting blood sugar, total cholesterol, triglycerides
Wang et al., 2019^50^	China	6 months	45.13	Hand-held clinic, monitoring prompts, dietary recommendations and exercise guidance via app	Diabetes knowledge, fasting blood sugar, HbA1c, self-efficacy
Zhang et al., 2019^51^	China	6 months	53	Smartphone app and online interactive management	BMI, fasting blood sugar, HbA1c
Lee et al., 2020^52^	Malaysia	52 weeks	53.3	Telemetry	Diabetes knowledge, fasting blood sugar, HbA1c, high-density lipoproteins, self-efficacy, total cholesterol, triglycerides
Owolabi et al., 2020^53^	South Africa	6 months	60.64	SMS for lifestyle advice and appointment reminders	Adherence to treatment
Wang et al., 2020^54^	Mongolia	12 months	55.4	SMS for health awareness, diet control, physical activities, living habits and weight control	Fasting blood sugar

app: application; BMI: body mass index; HbA1c: glycated haemoglobin; NR: not reported; SMS: short message service.

### Risk of bias 

Our assessment of the risk of bias revealed that 19 studies had a high risk of bias, while 12 studies had a low risk of bias. Within the studies with a low risk of bias, the highest number of individual matrices were found to have a low risk of bias across matrices of random sequence generation (24 matrices), followed by attrition bias (20 matrices), other risk of biases (10 matrices), allocation concealment (eight matrices) and blinding of outcome assessors (six matrices; data repository).^21^

### Intervention strategies 

Our included interventions varied in their strategies for the management of diabetes. We identified five modes of intervention delivery, through either smartphone apps (five studies), ^14^^,^^37^^,^^47^^,^^50^^,^^51^ SMS (nine studies), ^13^^,^^30^^,^^36^^,^^39^^,^^42^^,^^46^^,^^49^^,^^53^^,^^54^ telemetry (five studies),^32^^,^^38^^,^^40^^,^^48^^,^^52^ telephone (10 studies)^13^^,^^15^^,^^18^^,^^27^^,^^29^^,^^33^^,^^40^^,^^41^^,^^44^^,^^48^ and web-based systems including video conferencing (four studies).^31^^,^^35^^,^^43^^,^^45^ Most studies focused on a single mode of telemedicine delivery; however, one study considered both telephone and SMS and another study investigated the use of both telephone and telemetry.^13^^,^^40^ Major strategies included health record-keeping, follow-ups, reminders for follow-ups and logins, psychoeducation, glucose monitoring, monitoring prompts for serum glucose levels, pervasive alerts and online consultations (Table 1 and data repository).^21^ We did not observe any trend in the emergence of unique technologies; however, smartphone app-based interventions began to be tested from 2017. We provide details of individual interventions in the data repository.^21^

### Meta-analysis

**Fig. 2:**
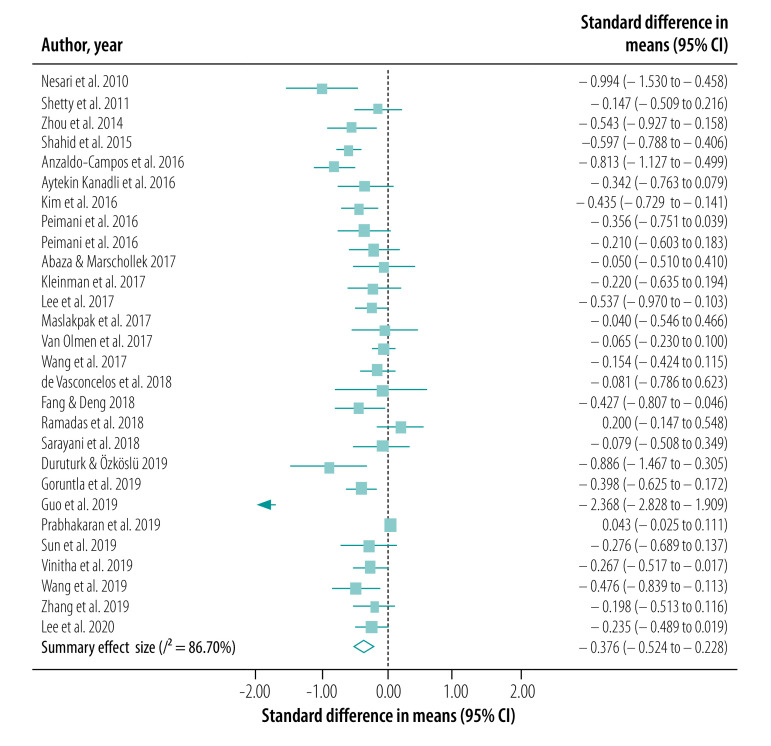
Forest plot showing effectiveness of telemedicine for diabetes treatment in improving serum HbA1c levels, low- and middle-income countries, 2010–2020

CI: confidence interval; HbA1c: glycated haemoglobin.

**Fig. 3:**
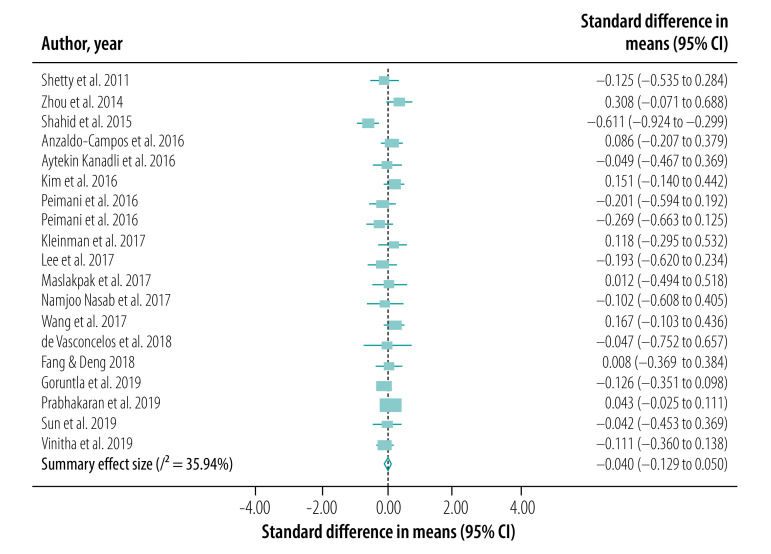
Forest plot showing effectiveness of telemedicine for diabetes treatment in improving body mass index, low- and middle-income countries, 2011–2019

**Fig. 4:**
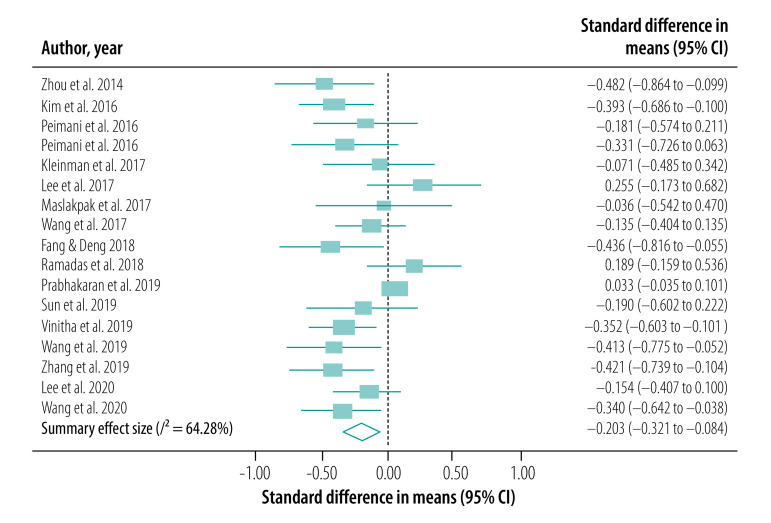
Forest plot showing effectiveness of telemedicine for diabetes treatment in improving fasting blood sugar levels, low- and middle-income countries, 2014–2020

**Fig. 5:**
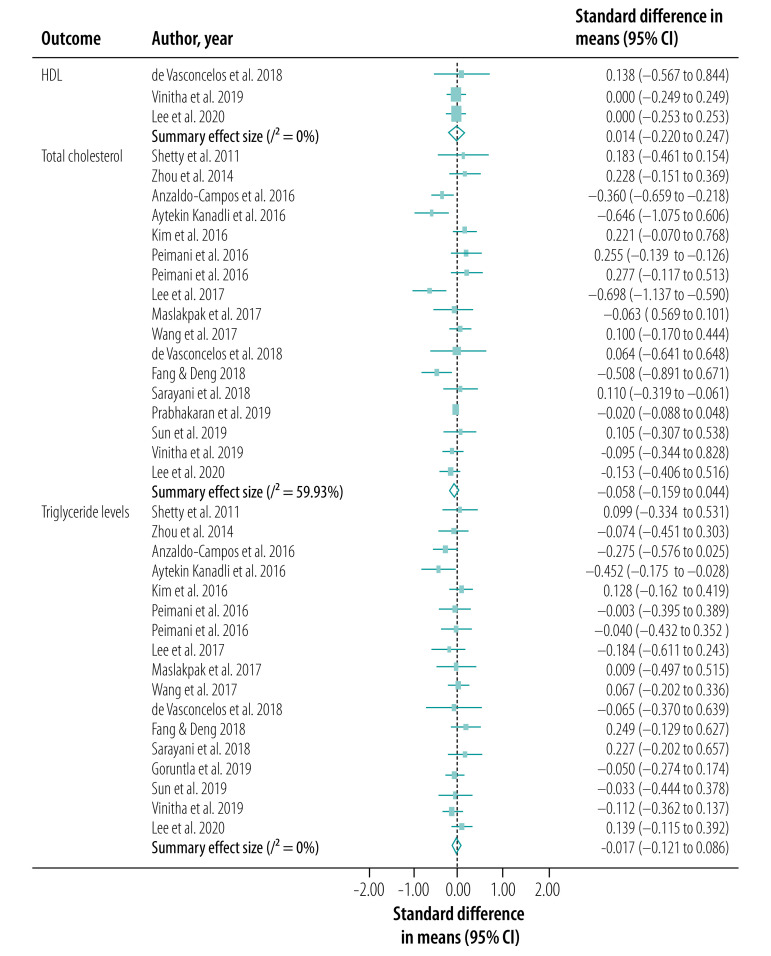
Forest plot showing effectiveness of telemedicine for diabetes treatment in improving serum lipid profile efficacy, low- and middle-income countries, 2011–2020

CI: confidence interval; HDL: high density lipoproteins.

**Fig. 6:**
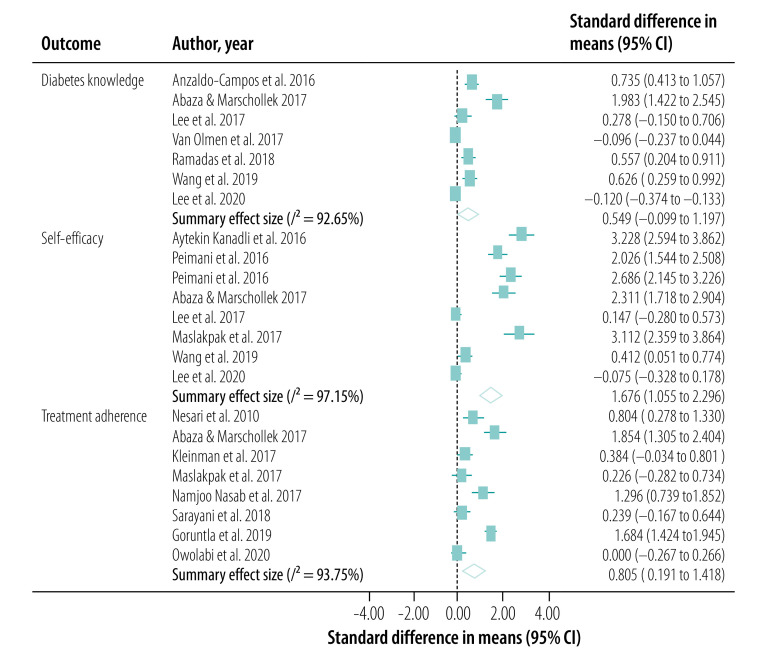
Forest plot showing effectiveness of telemedicine for diabetes treatment in improving adherence to treatment, diabetes knowledge and self-efficacy, low- and middle-income countries, 2010–2020

We conducted our meta-analysis of the 30 studies, including 31 interventions, across eight outcomes. Our primary outcome, HbA1c levels, was reported in a total of 27 studies (28 interventions; Fig. 2). BMI was reported in 18 studies (19 interventions; Fig. 3), serum levels of fasting blood sugar in 16 studies (17 interventions; Fig. 4), and total cholesterol and triglycerides in 16 studies (17 interventions; Fig. 5). Adherence to treatment was reported in eight studies (Fig. 6), knowledge of diabetes in seven studies (Fig. 6) and self-efficacy in seven studies (eight interventions; Fig. 6).

CI: confidence interval.

CI: confidence interval.

CI: confidence interval.

We observed a significant treatment effect among several outcomes, with standardized mean differences of −0.38 for HbA1c (95% CI: −0.52 to −0.23; *n* = 7703; *I*^2^ = 86.70%), −0.20 for fasting blood sugar (95% CI: −0.32 to −0.08; *n* = 5524; *I*^2^ = 64.28%), 0.81 for adherence to treatment (95% CI: 0.19 to 1.42; *n* = 959; *I*^2^ = 93.75%), 0.55 for knowledge of diabetes (95% CI: −0.10 to 1.20; *n* = 1585; *I*^2^ = 92.65%) and 1.68 for self-efficacy (95% CI: 1.06 to 2.30; *n* = 866; *I*^2^ = 97.15%). We did not observe any significant treatment effect in other outcomes, with standardized mean differences of −0.04 for BMI (95% CI: −0.13 to 0.05; *n* = 5957; *I*^2^ = 35.94%), −0.06 for total cholesterol (95% CI: −0.16 to 0.04; *n* = 5381; *I*^2^ = 59.93%) and −0.02 for triglycerides (95% CI: −0.12 to 0.09; *n* = 2360; *I*^2^ = 0%). 

Funnel plots and Egger regression analyses revealed that publication bias was significant for the outcomes of HbA1c, knowledge of diabetes, fasting blood sugar and self-efficacy (data repository).^21^ Publication bias was non-significant in outcomes including BMI, total cholesterol, triglycerides and adherence to treatment (data repository).^21^ Our sensitivity analysis did not reveal any significant changes in the effect sizes of these outcomes.

Our subgroup analysis based on mode of intervention delivery revealed that the only outcomes that yielded statistical significance were BMI and self-efficacy. Telephone- and SMS-based telemedicine interventions yielded the highest treatment effects when compared with telemetry and smartphone-based services for a range of outcomes (Table 2; available at: http://www.who.int/bulletin/volumes/99/3/19-250068). 

**Table 2:** Subgroup analysis of intervention delivery mode in the systematic review and meta-analysis of the effectiveness of telemedicine in the delivery of diabetes care, low- and middle-income countries, 2010–2020

Outcome and type of intervention	Standardized mean difference (95% CI)	*I*^2a^	*τ*^2a^	Between-group difference, *P*
**BMI**				0.002
Smartphone app	0.05 (−0.02 to 0.11)	0.00	0.00	
SMS	−0.13 (−0.26 to 0.00)	0.00	0.00	
Telemetry	0.05 (−0.11 to 0.22)	0.00	0.00	
Telephone	−0.27 (−0.47 to −0.07)	47.68	0.05	
Web-based	0.21 (−0.02 to 0.44)	0.00	0.00	
**Self-efficacy**				< 0.01
Smartphone app	0.41 (0.05 to 0.77)	0.00	0.00	
SMS	2.32 (2.01 to 2.62)	37.22	0.04	
Telemetry	−0.02 (−0.24 to 0.20)	0.00	0.00	
Telephone	3.18 (2.69 to 3.66)	0.00	0.00	
**Adherence to treatment**				0.67
Smartphone app	0.38 (−1.42 to 2.19)	0.00	0.00	
SMS	1.16 (0.13 to 2.20)	97.77	1.21	
Telephone	0.64 (−0.28 to 1.55)	74.15	0.18	
**Serum HbA1c levels**				0.66
Smartphone app	−0.58 (−0.96 to −0.20)	96.42	0.54	
SMS	−0.24 (−0.54 to 0.06)	14.16	0.00	
Telemetry	−0.40 (−0.78 to −0.02)	66.59	0.05	
Telephone	−0.37 (−0.74 to 0.00)	61.15	0.07	
Web-based (video conference)	−0.89 (−1.85 to 0.08)	0.00	0.00	
**Diabetes knowledge**				0.90
Smartphone app	0.63 (−1.02 to 2.27)	0.00	0.00	
SMS	0.89 (−0.28 to 2.05)	97.99	2.12	
Telemetry	0.30 (−0.65 to 1.24)	88.11	0.21	
Web-based	−0.26 (−0.75 to 0.23)	80.33	0.12	
**Fasting blood sugar levels**				0.56
Smartphone app	−0.17 (−0.39 to 0.04)	76.54	0.06	
SMS	−0.33 (−0.54 to −0.12)	0.00	0.00	
Telemetry	−0.08 (−0.31 to 0.15)	2.10	0.00	
Telephone	−0.04 (−0.64 to 0.56)	0.00	0.00	
Web-based	0.56 (−1.08 to 2.20)	0.00	0.00	
**Serum total cholesterol levels**				0.50
Smartphone app	−0.02 (−0.48 to 0.44)	0.00	0.00	
SMS	0.00 (−0.27 to 0.27)	63.77	0.07	
Telemetry	−0.18 (−0.44 to 0.07)	68.37	0.06	
Telephone	−0.16 (−0.50 to 0.18)	56.98	0.09	
**Serum triglyceride levels**				0.90
Smartphone app	0.22 (−0.18 to 0.63)	0.00	0.00	
SMS	−0.01 (−0.14 to 0.12)	0.00	0.00	
Telemetry	−0.03 (−0.17 to 0.12)	23.97	0.01	
Telephone	−0.08 (−0.33 to 0.17)	40.59	0.04	
Web-based	0.05 (−0.19 to 0.29)	0.00	0.00	

app: application; CI: confidence interval; HbA1c: glycated haemoglobin; SMS: short message service.

^a^ Both *I*^2^ and *τ*^2^ are measures of heterogeneity in subgroup analyses.

Our meta-regression analysis did not reveal any association between effect size and either quality assessment score or year of study for the HbA1c outcome (*P* > 0.05; data repository).^21^ We could not run a meta-regression analysis based on sex, mean age and time at which the measurement of outcomes was reported because of missing data.

### GRADE evidence

We assessed the certainty of evidence for the efficacy of telemedicine-based interventions for diabetes management in low- and middle-income countries, according to GRADE guidelines, for the critical outcome of HbA1c levels. We graded the certainty of evidence as very low because of substantial heterogeneity, publication bias and risk of bias (data repository).^21^

## Discussion

Our meta-analysis showing that telemedicine-based interventions are effective in improving serum levels of HbA1c and fasting blood sugar, adherence to treatment and self-efficacy is consistent with several individual RCTs conducted in both high-income countries and in low- and middle-income countries.^13^^,^^16^^,^^18^^,^^29^^,^^35^^,^^37^^,^^55^ Our results are also in accordance with previously conducted meta-analyses; for example, in the 2014 global study of the clinical effectiveness of telemedicine in reducing serum levels of HbA1c, researchers reported a small but statistically significant decrease (standardized mean difference: −0.37).^19^ Our results are also corroborated by the 2017 global systematic review, which showed moderate reductions in HbA1c post-intervention with reduced effect sizes at follow-up.^11^ Telemedicine interventions were also found to be cost-effective for diabetes management; for example, for retinal screening alone, telemedicine interventions were reported to yield 113.48–3828.46 quality-adjusted life years.^12^ Although an outcome of interest, we could not find any cost–effectiveness data applicable to low- and middle-income countries.

Despite the encouraging effect sizes for a range of outcomes, we found no improvement in BMI or serum levels of total cholesterol or triglycerides. It was not possible to corroborate these results as our literature review did not yield any similar meta-analytic reviews exploring these indicators. The statistical non-significance or poor treatment effects of these interventions for these outcomes can be explained, however. First, most of these interventions were developed to target single outcomes, such as adherence to treatment, HbA1c serum levels or dietary behaviour.^13^^,^^55^^,^^56^ None of the interventions focused on the measurement of lipid profile or BMI, and none assessed knowledge of diabetes (telemetric or otherwise). Second, the sample size calculations of these trials were based on improvement in HbA1c levels. We recommend that future interventions should be developed as comprehensive packages providing sessions on diet, physical exercise, monitoring of HbA1c levels and adherence to treatment.

For several outcomes, including BMI, adherence to treatment and self-efficacy, we found telephone- and SMS-based interventions (i.e. low-tech services) to be more effective than telemetry, smartphone apps or other web-based interventions. However, previous studies did not compare outcomes for different telemedicine delivery modes, meaning that we could not find any corroboratory or contradictory evidence. We suggest that our observed increased effectiveness of telephone- and SMS-based interventions may be attributable to the improved relationship between health-care professional and patient obtainable through these media.^57^ Importantly, the fact that these delivery modes performed as well as or better than more advanced apps and software means that they can be adopted with confidence in low-resource settings. 

Regarding other modes of delivery, smartphone apps developed in future studies should of course be user-friendly with a patient-centredness perspective, and considerate of the computer literacy levels of patients. Future researchers should consider conducting participatory approaches, including pilot surveys, design science, cost–effectiveness studies, and knowledge, attitude and practice surveys to explore levels of computer literacy among consumers.

Our review has several limitations. First, our search was limited to only five major academic databases and two minor grey-literature databases. Several additional databases, such as Embase and APA PsycINFO®, also warrant searching for potential articles. Although the databases Web of Science, PubMed® and Cochrane registry are highly inclusive, literature may have been missed. Our results are also limited by a high statistical and methodological heterogeneity across a few outcomes. For instance, outcomes such as self-efficacy and adherence to treatment were assessed using heterogeneous questionnaires. The heterogeneity in the outcomes could also be explained by different modes of intervention delivery, geographical regions and intervention contents.

Despite the above limitations, by synthesizing evidence from 31 studies conducted in low- and middle-income countries, we have provided more inclusive evidence in terms of number of articles than the previously published key reviews.^11^^,^^19^ Our study also benefited from the exploration of additional outcomes, including a variety of biochemical indicators. 

Our meta-analysis revealed a very low certainty of evidence that telemedicine interventions were effective in improving diabetes management in low- and middle-income countries. Higher-quality RCTs are required before a solid recommendation for the use of telemedicine-based interventions can be made. We recommend that future interventions should be designed to address both the biological and socioeconomic determinants of diabetes. Studies that explore the evaluation, feasibility or acceptability of data are important in the scaling up of interventions. 

To conclude, telemedicine-based services are frequently considered to be costly to the health system, but there should be more reviews addressing the cost–effectiveness of implementing and integrating such interventions within national health systems. Telemedicine would benefit from integration with the community-based health system with the support of community health workers. Despite barriers to this integration, telemedicine could improve the accessibility and quality of health-care services, improve personnel training and management processes, and optimize the use of epidemiological and clinical data.^58^
